# Podocytes Degrade Endocytosed Albumin Primarily in Lysosomes

**DOI:** 10.1371/journal.pone.0099771

**Published:** 2014-06-12

**Authors:** John M. Carson, Kayo Okamura, Hidefumi Wakashin, Kim McFann, Evgenia Dobrinskikh, Jeffrey B. Kopp, Judith Blaine

**Affiliations:** 1 University of Colorado Health Sciences Center, Aurora, Colorado, United States of America; 2 Kidney Disease Section, NIDDK, NIH, Bethesda, Maryland, United States of America; Fondazione IRCCS Ospedale Maggiore Policlinico & Fondazione D'Amico per la Ricerca sulle Malattie Renali, Italy

## Abstract

Albuminuria is a strong, independent predictor of chronic kidney disease progression. We hypothesize that podocyte processing of albumin via the lysosome may be an important determinant of podocyte injury and loss. A human urine derived podocyte-like epithelial cell (HUPEC) line was used for *in vitro* experiments. Albumin uptake was quantified by Western blot after loading HUPECs with fluorescein-labeled (FITC) albumin. Co-localization of albumin with lysosomes was determined by confocal microscopy. Albumin degradation was measured by quantifying FITC-albumin abundance in HUPEC lysates by Western blot. Degradation experiments were repeated using HUPECs treated with chloroquine, a lysosome inhibitor, or MG-132, a proteasome inhibitor. Lysosome activity was measured by fluorescence recovery after photo bleaching (FRAP). Cytokine production was measured by ELISA. Cell death was determined by trypan blue staining. *In vivo*, staining with lysosome-associated membrane protein-1 (LAMP-1) was performed on tissue from a Denys-Drash trangenic mouse model of nephrotic syndrome. HUPECs endocytosed albumin, which co-localized with lysosomes. Choloroquine, but not MG-132, inhibited albumin degradation, indicating that degradation occurs in lysosomes. Cathepsin B activity, measured by FRAP, significantly decreased in HUPECs exposed to albumin (12.5% of activity in controls) and chloroquine (12.8%), and declined further with exposure to albumin plus chloroquine (8.2%, p<0.05). Cytokine production and cell death were significantly increased in HUPECs exposed to albumin and chloroquine alone, and these effects were potentiated by exposure to albumin plus chloroquine. Compared to wild-type mice, glomerular staining of LAMP-1 was significantly increased in Denys-Drash mice and appeared to be most prominent in podocytes. These data suggest lysosomes are involved in the processing of endocytosed albumin in podocytes, and lysosomal dysfunction may contribute to podocyte injury and glomerulosclerosis in albuminuric diseases. Modifiers of lysosomal activity may have therapeutic potential in slowing the progression of glomerulosclerosis by enhancing the ability of podocytes to process and degrade albumin.

## Introduction

Albuminuria is a common feature of many kidney diseases and independently predicts kidney disease progression [1], [2], [3], [4]. Albuminuria is not only a result of kidney disease, it perpetuates progressive renal damage [1]. Most of the research involving the toxic effects of albumin has focused on the tubulointerstitium. The effect of albumin on podocytes has not been thoroughly investigated. Previous studies have shown that endocytosed albumin induces pro-inflammatory cytokines [5], [6], endoplasmic reticulum (ER) stress [7], and apoptosis [8] in podocytes. However, how podocytes process endocytosed albumin and the mechanisms by which albumin induces inflammation, ER stress, and apoptosis have not been defined.

Filtration of albumin into the urine is limited by the size and charge selectivity of the glomerular filtration barrier (GFB), which consists of fenestrated, glycocalyx-rich endothelial cells, the glomerular basement membrane, and podocytes [9]. The mechanism by which albumin and other macromolecules are filtered by the glomerulus has been an area of active research for years [10], [11]. More recently, the amount of albumin that passes through the GFB has been a matter of debate [12], [13]. The most widely accepted view is that little albumin crosses the glomerular filtration barrier (GFB) in individuals with normal renal function [10], [11]. Nevertheless, taking into account even the most conservative estimates of the fractional clearance of albumin (0.062%), normal human kidneys filter more than 3 grams of albumin daily [14]. The proximal tubule reabsorbs nearly the entire amount of filtered albumin.

Traditionally podocytes are thought to serve a sieving function through their inter-digitating foot processes connected by slit diaphragms. Proof of this function is supported by the massive proteinuria that results from mutations involving slit diaphragm proteins such as nephrin and podocin [15], [16]. However, the function of podocytes is not limited to sieving. Studies demonstrate both human and animal podocytes endocytose albumin *in vitro* [17], [18]. Data also suggests that podocytes clear proteins from the GBM via transcytosis [19]. These studies suggest that, under normal physiologic conditions, podocytes internalize albumin and other plasma proteins (e.g. IgG) to prevent the GFB from clogging. Increased protein filtration, via disruption of the endothelial cell layer or GBM, has the potential to overwhelm the capacity of podocytes to clear and process proteins. The presence of protein resorption droplets within podocytes, as demonstrated in biopsies from nephrotic humans and animal models of nephrosis [20], [21], [22], suggests an inability of podocytes to effectively process proteins.

Many eukaryotic cells selectively internalize specific macromolecules via receptor-mediated endocytosis in which clathrin or non-clathrin coated pits bud from the plasma membrane and fuse with early endosomes [23]. The early endosomes are sorting organelles that determine whether endocytosed molecules are recycled back to the plasma membrane, termed transcytosis, or trafficked to the lysosome for degradation [23]. In other cell types, such as proximal tubule cells, endocytosed albumin may be either transcytosed or degraded [24]. The molecule responsible for transcytosis in proximal tubule cells is the neonatal Fc receptor (FcRn), which diverts albumin from the degradative pathway allowing it to be reabsorbed at the basolateral membrane [25]. FcRn is expressed in podocytes [26] and we have found that cultured podocytes transcytose albumin [27].The relative contribution, however, of the transcytotic and degradative pathways to albumin processing in podocytes is unknown.

Here we examine the role of the lysosome in albumin degradation. We also determine whether lysosomal inhibition potentiates albumin toxicity. In a previous study we found pro-inflammatory cytokine production, cell death, and apoptosis are all increased in podocytes treated with albumin. In addition, albumin sequestration within podocytes is associated with podocyte loss and glomerulosclerosis [22]. This suggests that how podocytes take up and degrade albumin may be important determinants of podocyte cell death and loss of kidney function.

In this study, we utilized human urine derived epithelial cells (HUPECs) from subjects with normal renal function [28]. HUPECs express several podocyte markers and have been shown to behave similarly to other podocyte cell lines, including those derived from a human biopsy specimen [6]. Urine- derived podocytes are advantageous in that once the pathways for albumin processing have been defined in normal subjects, these can be examined in subjects with various glomerular diseases. Here we confirmed that endocytosed albumin in podocytes was degraded by lysosomes. We found that inhibiting lysosomes resulted in albumin accumulation in podocytes and potentiated albumin toxicity. To evaluate lysosome function in living podocytes in real time, we used fluorescence recovery after photobleaching (FRAP). To confirm our in vitro findings, we used a mouse model of nephrotic disease and showed that there was lysosomal upregulation.

## Materials and Methods

### Reagents

Human albumin conjugated to fluorescein isothiocyanate (FITC-albumin) was purchased from Cappel Laboratories (Malvern, PA). Recombinant, low-endotoxin human albumin was purchased from Sigma-Aldrich (St. Louis, MO). This albumin is fatty acid and globulin free. Using Limulus Amebocyte Lysate assay (Lonza, Walkersville, MD) we confirmed that the levels of endotoxin in this reagent are 0.005 EU endotoxin/µg albumin (considered endotoxin-free by industry standards). Chloroquine diphosphate salt was purchased from Sigma-Aldrich and dissolved in nanopure water to produce a 10 mM stock solution. The concentration of chloroquine used to treat cells in all experiments was 50 µM [29]. MG-132 (10 mM) solution was purchased from Sigma-Aldrich. The concentration used to treat cells in all experiments was 10 µM [30].

### Ethics Statement

Animal experiments were performed under a protocol approved in advance by the NIH Animal Care and Use Committee. Animal care adhered to the NIH Guidelines for the Care and Use of Laboratory animals (National Institutes of Health, Bethesda, MD). Human urine derived podocyte-like epithelial cells (HUPECs or podocytes) were isolated from the urine of patients who gave written informed consent under a protocol approved by the National Institute of Diabetes and Digestive and Kidney Diseases Institutional Review Board.

### Animals

The Denys-Drash syndrome mice carry a single copy of the Wt1 Arg394Trp mutation, and are maintained on an FVB/n background; these were the generous gift of Dr. Laurence Heidet (Universite Rene Descartes, Paris, France)[31]. We chose this model of nephrotic disease because it does not involve toxins such as adriamycin or lipopolysaccharide to induce albuminuria. As a result, the effects of albuminuria itself could be evaluated without the confounding effects of a podocyte toxin. Animal experiments were performed under a protocol approved in advance by the NIH Animal Care and Use Committee. Animal care adhered to the NIH Guidelines for the Care and Use of Laboratory animals.

### Cell culture

Human urine derived podocyte-like epithelial cells (HUPECs, termed podocytes for simplicity) isolated from a healthy volunteer were described previously [28]. The podocytes carried a temperature-sensitive variant of the simian virus (SV40) large tumor antigen and were cultured and maintained, as previously described [28]. Briefly, podocytes were grown on type I collagen-coated flasks (Fisher Scientific, Rochester, NY) at 33°C (growth permissive conditions) in culture medium (RPMI 1640 medium (Sigma), 10% fetal bovine serum (Hyclone, Logan, UT), 15 mM HEPES, 5 mM L-glutamine, 100 U/ml penicillin, 100 U/ml streptomycin; 5% CO_2_ atmosphere). After reaching confluency, cells were seeded onto 35 mm type I collagen-coated dishes and maintained at 37°C (growth restrictive conditions) for 7–12 days to allow for differentiation. Under these conditions, the cells express podocyte markers including synaptopodin, nestin, and CD2AP. All cells were used between passages 17 and 25.

### FITC-Albumin and Lysosome Co-localization

Briefly, podocytes were grown on collagen-coated dishes with cover slip bottoms (MatTek). Podocytes were treated with 1.5 mg/ml FITC-albumin in 1X Magic Red Cathepsin B solution for 1 hour. After 1 hour, the podocytes were thoroughly rinsed with PBS then immediately visualized using confocal microscopy. FITC-albumin was visualized using a 488 nm excitation laser and Magic Red Cathepsin B was visualized using a 633 nm excitation laser. The single channel images were merged to demonstrate co-localization of endocytosed albumin and lysosomes.

### FITC-Albumin Degradation

FITC-labeled albumin was used to monitor and measure the uptake and degradation of albumin by cultured podocytes. Podocytes were plated at a density of 2.8×10^4^ cells per dish in 35 mm collagen coated dishes (MatTek Corporation, Ashland, MA) and allowed to differentiate for 7–12 days. Eighteen hours prior to the experimental procedures, the media was changed to FBS-free media with or without 50 µM chloroquine or 10 µM MG-132.

For albumin degradation experiments, podocytes were incubated with 1.5 mg/ml human FITC-albumin solution for 60 min at 37°C. After 1 hour of loading, the podocytes were washed 6 times with ice cold PBS and incubated in Ringers solution. In the first degradation experiment, the time course of albumin degradation was determined by measuring abundance of FITC-albumin at times zero, 15, 30, 45, and 60 minutes after incubation in Ringer's solution. In the second degradation experiment, each group was run either with or without 50 µM chloroquine, an inhibitor of lysosomal protein degradation, or 10 µM MG-132, an inhibitor of proteasomal protein degradation. Chloroquine and MG-132 were added 18 hours prior to FITC-albumin loading and were present throughout the experiment. The inhibitors were added 18 hrs in advance to ensure adequate lysosomal and proteasomal inhibition prior to starting the degradation experiment. After FITC-albumin loading, podocytes were washed 6 times with ice-cold PBS and Ringer solution, with or without 50 µM chloroquine or 10 µM MG-132, was added for 0, 30 min and 60 min. For Western blot analyses, podocytes were lysed in 50 µl of 5xPAGE (50 mM Tris-base, 5% sodium dodecyl sulfate, 25% sucrose, 5 mM EDTA; pH 8.0) with Mini-Complete protease inhibitors (Roche; Indianapolis, IN) added. The abundance of FITC-albumin in the podocyte lysates was measured by Western blotting.

### Lysosome visualization

A Magic Red Cathepsin B assay kit was used to visualize lysosomes in living cells as described by Pryor [32]. Briefly, podocytes were grown on collagen-coated dishes with coverslip bottoms (MatTek). Podocytes were exposed to culture media alone (control), 50 µM chloroquine alone in culture media for 24 hours, 5 mg/ml low endotoxin human albumin alone in media for 24 hours, or 50 µM chloroquine and 5 mg/ml low endotoxin albumin in media for 24 hours. At 24 hours, the media was removed and the podocytes were incubated for 30 minutes in 260 µL of 1X Magic Red solution made with Magic Red substrate and culture media per the manufacturer's instructions. After 30 minutes, the Magic Red solution was removed, podocytes were rinsed with PBS and incubated in phenol-free media for visualization. Lysosomes within the podocytes were visualized by confocal microscopy using a 633 nm excitation laser. Because lysosomes are three-dimensional structures, quantifying lysosome size using two-dimensional imaging is limited. Due to this limitation, we quantified lysosomes by measuring the density of Magic Red fluorescence within each cell at a plane in which the lysosomes were most prominent. Fluorescence images were obtained using Zeiss laser-scanning confocal/multiphoton-excitation fluorescence microscope with a Meta spectral detection system (Zeiss NLO 510 with META, Zeiss, Thornwood, NY). The imaging settings were initially defined empirically to maximize the signal to noise ratio and to avoid saturation. In comparative imaging, the settings were kept constant between samples. We quantified lysosome density by normalizing the Magic Red immunofluorescence intensity to the podocyte cell area.

### Lysosome activity

Fluorescence recovery after photobleaching (FRAP) experiments were performed to determine active cathepsin B activity in live cells as described by Pryor [32]. Briefly, podocytes were grown on collagen coated dishes with coverslip bottoms (MatTek). Cells were exposed to culture media alone (controls), 50 µM chloroquine alone in media, 5 mg/ml low endotoxin human albumin alone in media, or 50 µM chloroquine and 5 mg/ml low endotoxin albumin in media for 24 hours. At 24 hours, podocytes were incubated for 30 minutes in Magic Red as described in the previous paragraph. During visualization of podocytes, a region of interest (ROI) containing a single lysosome was defined. Three images of the ROI were taken prior to bleaching. The ROI was bleached with 100 iterations using 488, 543, and 633 nm lasers for approximately 10 seconds to reduce the ROI fluorescence intensity to at least 50% of the baseline intensity. The ROI was then continuously scanned (scan time approximately 500 ms) until the fluorescence in the ROI recovered to a plateau. Fluorescence intensity relative to peak intensity (y-axis) was plotted versus time (x-axis). A nonlinear regression curve with one-phase decay was fit to the data enabling determination of half-time (t_½_) of recovery using GraphPad Prism 5 software (LaJolla, CA).

### Western Blotting

For podocytes lysed in a 5xPAGE buffer, protein concentrations were measured by BCA assay (Pierce; Rockford, IL) and samples were reduced (10% β-mercaptoethanol). Cell lysates were run on 9% polyacrylamide gels and transferred onto nitrocellulose membranes (Bio-Rad, Hercules, CA). Subsequent blocking, antibody and wash solutions were diluted in blot buffer (150 mM NaCl, 10 mM Na_2_HPO_4_, 5 mM EDTA, 1% Triton X-100; pH 7.4). Membranes were initially blocked (5% non-fat dry milk; 60 min) and then incubated with primary antibody. Primary antibodies include: FITC (1∶1,000; Santa Cruz Biotech; Santa Cruz, CA) and GAPDH (1∶1,000; Santa Cruz Biotech). Blots were then washed, incubated with horseradish peroxidase-conjugated secondary antibodies (1∶10,000 dilution; Jackson ImmunoResearch, West Grove, PA) and washed. The antibody complexes were detected using enhanced chemiluminescence (Pierce) and captured using a photodocumentation system (UVP; Upland, CA). All densitometry measurements were normalized first to a loading control, GAPDH. For the degradation experiments, after normalizing for GAPDH, the albumin abundance was normalized to time zero.

### ELISA

Podocytes were grown on 35 mm collagen coated dishes as described above. Cells were exposed to culture media alone (control), 50 µM chloroquine alone in media, 5 mg/ml low endotoxin human albumin alone in media, or 50 µM chloroquine and 5 mg/ml low endotoxin albumin in media for 1, 3, or 18 hours. At the time of harvest, the cell supernatant was collected and the amount of cytokine present was analyzed using IL-6, TNF, or IL-1β ELISA kits (Biolegend, San Diego, CA). The podocytes in each dish were harvested in ice-cold (RIPA) buffer (50 mM Tris HCl, pH 8, 150 mM NaCl, 1% NP-40, 0.5% sodium deoxycholate, 0.1% SDS) with Mini-Complete protease inhibitors added (Roche). Cytokine amount was normalized to total protein content as determined by the BCA assay.

### Cell Death

Cell death was measured using the Trypan blue exclusion assay and an automated Cellometer Auto T4 Cell Counter Version 3.1.1 (Nexcelom Bioscience, Lawrence, MA). Cells were exposed to culture media alone, 50 µM chloroquine alone in media, 5 mg/ml low endotoxin human albumin alone in media, or 50 µM chloroquine and 5 mg/ml low endotoxin albumin in media for 24 hours. The cells to be counted were trypsinized and collected in a microfuge tube. The cellular suspension was centrifuged at 1000 *g* for 5 minutes, the pellet was washed once with phosphate buffered saline (PBS) and re-suspended in a small volume of PBS. An aliquot of cell suspension was then incubated for 3 minutes with an equal volume of 0.4% trypan blue solution [33]. Living and dead cells were identified and counted using a Cellometer. All experiments were performed at least three times.

### Histology

Denys-Drash mice were euthanized at 4 weeks of age, and kidneys were fixed in zinc formalin, embedded in paraffin, sectioned at 4 µm, and stained with periodic acid- Schiff. For immunofluorescence studies, kidneys were placed in cryoembedding compound and sectioned at 3 µm. Kidney sections were deparaffinized in xylene then hydrated in ethanol. After rinsing in distilled water, the sections were immersed in sodium citrate buffer at 95–100°C for 30 minutes. After cooling, sections were blocked (10% normal goat serum and 1% BSA in PBS with 0.1% Triton X) and incubated overnight at 4°C with primary antibody (LAMP-1 (1∶100; Santa Cruz Biotech). Primary antibody was not applied to one ‘no-primary control’ section. After washing, the sections were incubated (60 min, room temperature) with appropriate mix of Alexa 488-conjugated goat anti-rat IgG (1∶250; Invitrogen, Carlsbad, CA) and Alexa 635-conjugated phalloidin (1∶250; Invitrogen). Sections were then washed with PBS and mounted in Vectashield with DAPI (Vector Laboratories, Burlingame, CA). Fluorescence images were obtained using Zeiss laser-scanning confocal/multiphoton-excitation fluorescence microscope with a Meta spectral detection system (Zeiss NLO 510 with META, Zeiss, Thornwood, NY). The imaging settings were initially defined empirically to maximize the signal to noise ratio and to avoid saturation. In comparative imaging, the settings were kept constant between samples. Series of confocal fluorescence images were simultaneously obtained with a Zeiss C-Apochromat 40x/1.2 NA water objective using the 488 nm, 543 nm, and 633 nm laser lines for excitation.

### Albumin- Creatinine Ratio

Mouse urine was obtained during the day and urine albumin and creatinine were measured by ELISA (Exocell, Philadelphia, PA).

### Statistical Analysis

All data are presented as mean ±SEM. Urine albumin creatinine data were analyzed by Mann-Whitney test. The cytokine experiments were analyzed using a two-way ANOVA, with treatment group and time as the independent variables, and a Tukey-Kramer post test. All other differences between groups were analyzed using a one-way ANOVA with Dunnett's post test for more than two groups. P<0.05 was determined to be statistically significant.

## Results

### Endocytosed albumin co-localized with lysosomes and was degraded in lysosomes

**Figure 1:**
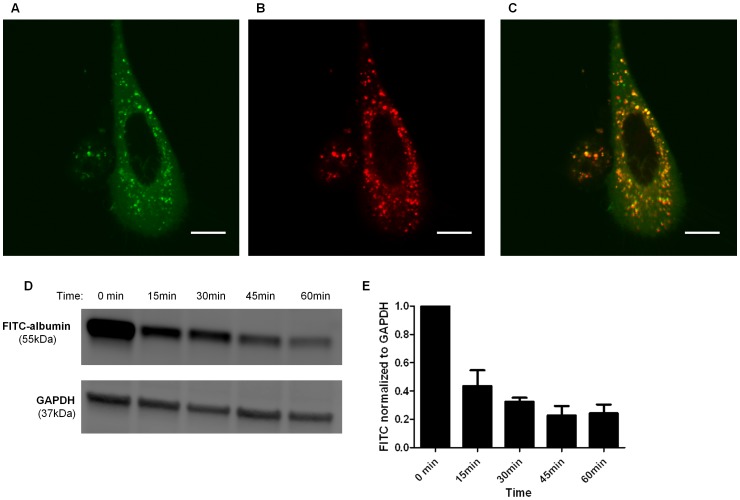
Endocytosed albumin co-localized with lysosomes and was degraded in podocytes. Confocal microscopy image demonstrating co-localization of albumin and lysosomes in a podocyte treated with 1.5 mg/mL FITC-albumin and Cathepsin B Magic Red for 1 hour. FITC- albumin was excited with a 488 nm laser and Magic Red with a 633 nm laser. FITC-Albumin is labeled green (**A**) and Magic Red, which selectively localizes in lysosomes, is labeled red (**B**). Yellow fluorescence represents albumin and Magic Red co-localization (**C**). Scale bar is 20 µm. **D**) Representative Western blot demonstrating the time course of albumin degradation. Podocytes were treated with 1.5 mg/mL FITC-albumin for 1 h then washed. Cells were harvested at time zero, 15, 30, 45, and 60 min. Albumin abundance in cell lysates was quantified by Western blot analysis using an antibody to FITC. **E**) Densitometry demonstrating an exponential decrease in abundance of FITC-albumin over 60 minutes normalized to GAPDH and abundance at time zero (n = 4).

Podocytes have been shown to endocytose albumin [6], [34] but the fate of endocytosed albumin in podocytes remains unknown. In order to identify lysosomes in living podocytes in real time, we used Cathepsin B Magic Red, a substrate that easily penetrates the cell membrane and membranes of internal cellular organelles in a non-fluorescent state and localizes to acidic organelles such as lysosomes. In the presence of active cathepsin B, the Cathepsin B Magic Red is cleaved and the cresyl violet fluorophore fluoresces upon excitation. Because cathepsin B is only active in the acidic pH of lysosomes, Cathepsin B Magic Red is an excellent lysosomal marker in living cells. To examine the co-localizaion of albumin and lysosomes in podocytes, podocytes were treated with 1.5 mg/mL FITC-albumin in 1X Magic Red Cathepsin B solution for 1 hr, rinsed, then imaged by confocal microscopy. **Figures 1A and 1B** show FITC-albumin (green) and lysosomes labeled with Cathepsin B Magic Red, respectively, located within the podocyte cytoplasm. Co-localization of albumin and Magic Red is demonstrated by the yellow vesicles in **Figure 1C**. To further examine albumin disposal in podocytes, cells were incubated with 1.5 mg/ml FITC albumin for one hour, rinsed very well and then harvested immediately (t = 0) and at 15, 30, 45, and 60 minutes. Albumin abundance in lysates was measured by Western blot (**Figure 1D**). Densitometric analysis of cell lysates (**Figure 1E**) showed that FITC-albumin abundance declined exponentially over 60 minutes. The albumin abundance at each time point represents the amount of albumin remaining within the cell but does not distinguish between albumin degradation within the cell or possible transcytosis through the cell.

### Albumin degradation in podocytes decreased with lysosome inhibition, but not proteasome inhibition

**Figure 2:**
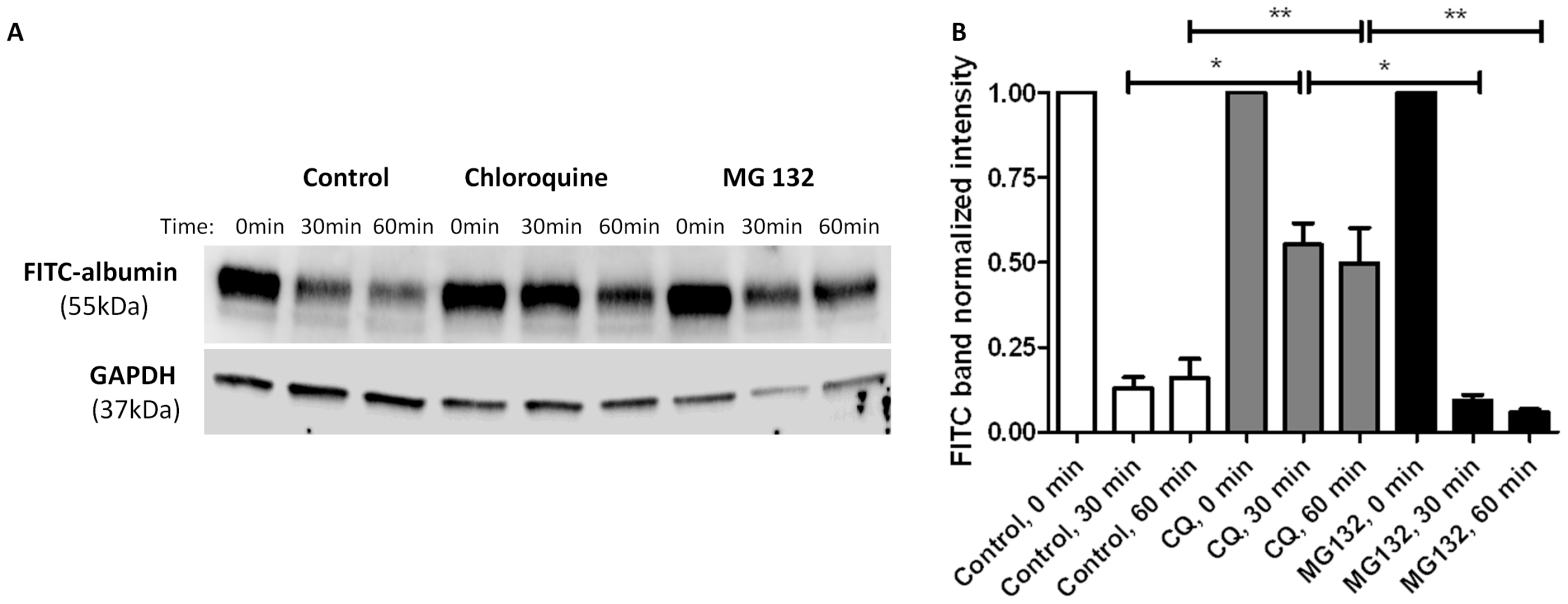
Albumin degradation in podocytes decreased with lysosome inhibition, but not proteasome inhibition. Densitometry and representative Western blot demonstrating albumin degradation. Podocytes were pretreated with standard media (controls, n = 7), 50 µM chloroquine (lysosome inhibitor, n = 5), or 10 µM MG-132 (proteasome inhibitor, n = 4) for 24 h. At 24 h, podocytes were treated with 1.5 mg/mL FITC-albumin for 1 h then washed. Cells were harvested at time zero, 30 min, and 60 min after washing. **A**) Albumin abundance in cell lysates was quantified by Western blot analysis using an antibody to FITC. **B**) Albumin abundance in chloroquine treated cells was significantly increased compared to control and MG-132 treated cells at 30 min (*p<0.05) and 60 min (**p<0.05). Densitometry was normalized to GAPDH and time zero.

Most endocytosed proteins that are targeted for degradation are sent to the lysosome whereas aberrant endogenous proteins are degraded within the proteasome. Whether podocytes degrade albumin in lysosomes or proteasomes is unknown. To examine this, podocytes were first treated with Ringers solution (control), 50 µM chloroquine, a lysosomal inhibitor, or 10 µM MG-132, a proteasomal inhibitor, and then were loaded with 1.5 mg/ml FITC-albumin for 1 hr. After loading, the podocytes were rinsed very well and then harvested immediately (t = 0) or at 30 min and 60 min. FITC-albumin abundance in lysates was measured by Western blot (**Figure 2A**). FITC-albumin abundance in lysates from control podocytes at times 30 and 60 min was 12.9±3.4% and 16.0±5.5%, respectively, relative to FITC-albumin abundance at t = 0. FITC-albumin abundance in lysates of chloroquine treated podocytes at times 30 and 60 min was 55.2±6.3% and 49.8±1.0%, respectively, relative to FITC-albumin abundance at t = 0. FITC-albumin abundance in lysates from MG-132 treated podocytes at times 30 and 60 min was 9.3±1.8% and 6.0±0.7%, respectively, relative to FITC-albumin abundance at t = 0. Densitometric analysis of cell lysates shows that FITC-albumin abundance in chloroquine treated podocytes at 30 and 60 minutes was significantly higher than that of MG-132 treated and control podocytes at 30 and 60 min (P<0.05 compared to MG-132 and control, (**Figure 2B**). The increased abundance of FITC-albumin in chloroquine treated podocytes indicates that lysosomal inhibition decreases albumin degradation in podocytes. FITC-albumin abundance in MG-132 treated podocytes at 30 and 60 min was not significantly different than FITC-albumin abundance in control podocytes at 30 and 60 min. Therefore, proteasome inhibition does not appear to decrease albumin degradation in podocytes. Lysosomal processing of albumin in podocytes is further supported by data demonstrating inhibition of albumin degradation by leupeptin, another lysosomal inhibitor, recently published elsewhere (**Data S1**) [27].

### Exposure of podocytes to albumin and chloroquine increases lysosome density in podocytes

**Figure 3:**
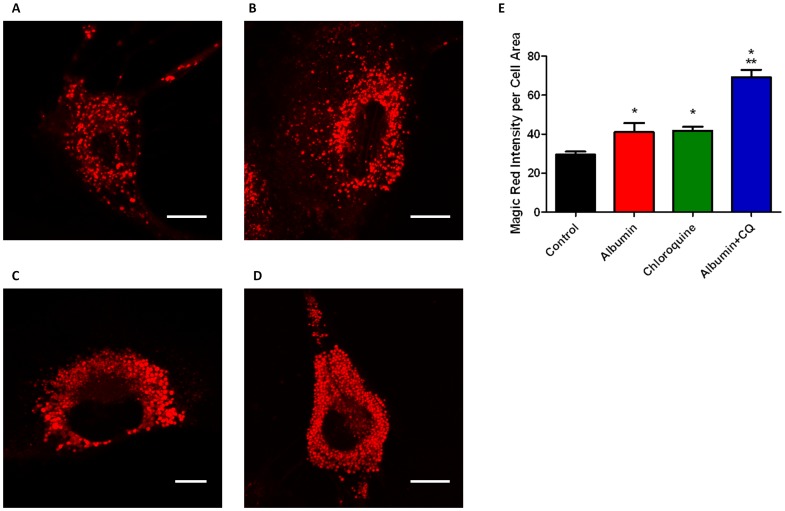
Exposure of podocytes to albumin and chloroquine increased lysosome fluorescence. Representative individual podocytes are shown for **A**) negative control **B**) albumin (5 mg/mL) exposure for 24 h **C**) chloroquine (50 µM) exposure for 24 h **D**) albumin (5 mg/mL) + chloroquine (50 µM) exposure for 24 h. After treatment with Magic Red Cathepsin B, individual lysosomes fluoresce red in the presence of active cathepsin B. **E**),Exposure to albumin and chloroquine increased the density of lysosome fluorescence. The effect was potentiated in podocytes exposed to both albumin and chloroquine. Fluorescence is expressed in arbitrary units. Scale bar is 20 µm. (n = 8 for all treatment groups, * p<0.05 vs control. **p<0.05 vs albumin or chloroquine alone).

In order to examine lysosomal density in living podocytes in real time, we used the Cathepsin B Magic Red fluorophore. One of the utilities of this substrate is to provide an assessment of the number and size of lysosomes within living cells. Because lysosomes are three-dimensional structures, quantifying lysosome size using two-dimensional imaging is limited. Due to this limitation, we quantified the lysosomes by measuring the density of Magic Red fluorescence within each cell at a plane in which the lysosomes were most prominent. We quantified lysosome density by normalizing the Magic Red immunofluorescence intensity to the podocyte cell area. Podocytes exposed to albumin (**Figure 3B**) or chloroquine (**Figure 3C**) showed increases in the density of Magic Red fluorescence compared to untreated control podocytes (**Figure 3A**). Exposure to both albumin and chloroquine combined (**Figure 3D**) resulted in a significant increase in the density of Magic Red immunofluroescence within podocytes, compared to treatment with either albumin or chloroquine alone. Magic Red Cathepsin B fluorescence was measured using Image J and normalized to cell area to quantify the density of lysosome fluorescence (**Figure 3E**, n = 8 for each exposure * p<0.05 vs Control, ** p<0.05 vs albumin and chloroquine alone).

### Prolonged exposure of podocytes to albumin and chloroquine decreased lysosome activity

**Figure 4:**
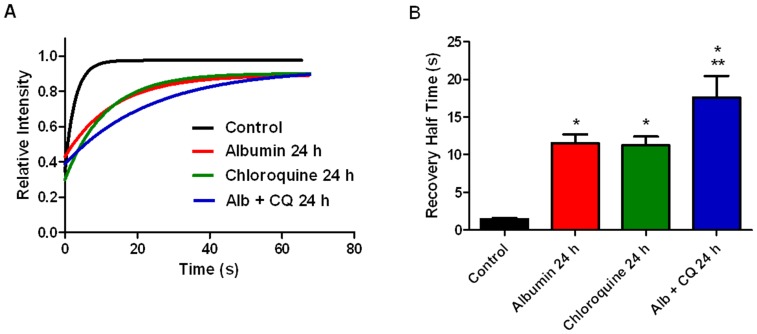
Prolonged exposure of podocytes to albumin and chloroquine decreased lysosome activity. Lysosome activity in living podocytes was determined by fluorescence recovery after photobleaching (FRAP). Using confocal microscopy, regions of interest containing individual lysosomes were isolated and photobleached. Exponential curves of the recovery of fluorescence intensity, which represents cathepsin B activity, were obtained. **A**) Representative recovery curves for control (black), 24 h albumin exposed (red), 24 h chloroquine exposed (green), and podocytes exposed to both albumin and chloroquine for 24 h (blue). **B**) Bar graph of recovery half times (mean +/− SEM), which are inversely related to cathepsin B activity. Cathepsin B activity was decreased in 24 h chloroquine (n = 10) and 24 h albumin (n = 10) exposed podocytes compared to control (n = 10), *p<0.05. Cathepsin B activity decreased further in podocytes exposed to both albumin and chloroquine for 24 h (n = 10) compared to each exposure alone, **p<0.05.

Cathepsin B Magic Red can also be used to directly measure cathepsin B activity within a living cell [32]. Activity of cathepsin B, a lysosomal cysteine protease, corresponds with overall lysosome activity. In addition, cathepsin B has been shown to be involved in albumin degradation in the kidney [35]. Fluorescence recovery after photobleaching (FRAP) was used to compare active cathepsin B activities in living podocytes under various treatment conditions. Lysosomes in podocytes were labeled with Cathepsin B Magic Red which is a fluorophore conjugated to a substrate specific for cathepsin B. Cleavage of the substrate by cathepsin B in the lysosome leads to the release of the fluorophore which fluoresces when excited. To perform FRAP, a region of interest (ROI) encompassing an individual lysosome was selected. The Magic Red signal was photobleached and the time taken for fluorescence recovery was measured. The intensities graphed are relative to the fluorescence intensity pre-bleach. Representative curves for each condition are shown in **Figure 4A**. The half-time (t½) of fluorescence recovery is proportional, and indirectly related, to cathepsin B activity and thus lysosome activity. Compared to control podocytes (black curve and bar) the recovery t½ was significantly prolonged in podocytes treated with 5 mg/ml albumin for 24 hours (red curve and bar), 1.4±0.2 vs. 11.6±1.2 seconds (p<0.05). The prolonged t½ reflects a decrease in lysosome activity. Albumin, like the Magic Red reagent, is a substrate for cathepsin B. Therefore, the prolonged t½ with albumin treatment may represent either a competitive effect of the albumin for cathepsin B, a decrease in cathepsin B activity, or both. By whichever mechanism albumin prolonged t½, the data suggest that endocytosed albumin affects lysosomal activity.Chloroquine inhibits lysosomal activity by raising pH.Therefore, we expected the t½ for fluorescence recovery to be prolonged in podocytes treated with chloroquine. As expected, lysosome activity was significantly increased in podocytes exposed to 50 µM chloroquine (green curve and bar) compared to controls 1.4±0.2 vs. 11.2±1.2 seconds (p>0.05). The t½ for recovery was significantly prolonged in podocytes treated with 5 mg/ml albumin plus 50 µM chloroquine (blue curve and bar) compared to albumin alone and chloroquine alone, 17.6±2.9 vs. 11.6±1.2 and 11.2±1.2 seconds (p<0.05). Therefore, exposure to albumin and chloroquine combined significantly decreased lysosome activity compared to controls and either albumin or chloroquine alone (p<0.05). This suggests albumin and chloroquine have an additive effect on cathepsin B activity.

### Chloroquine potentiated the effect of albumin on increased cytokine production in podocytes

**Figure 5:**
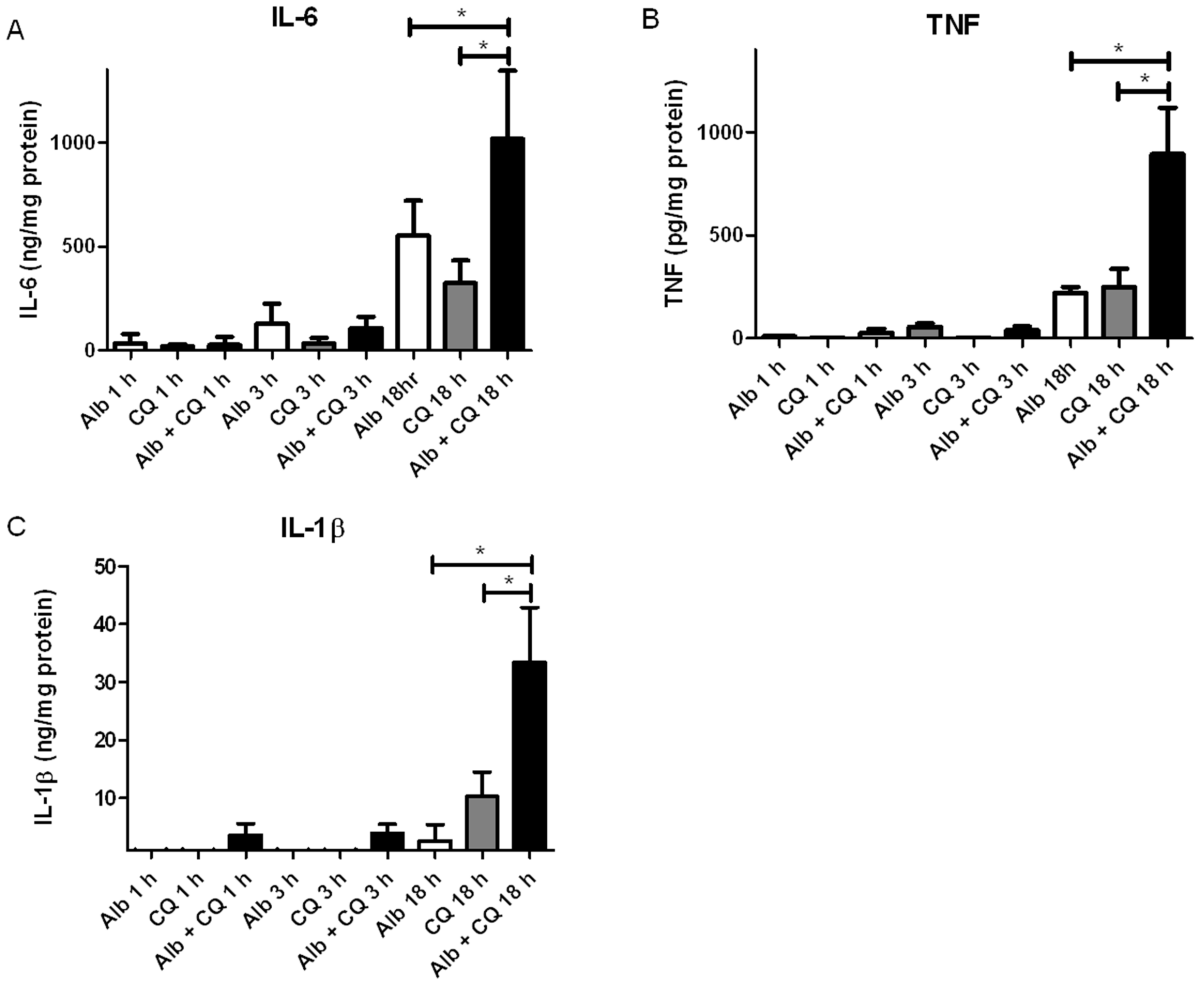
Chloroquine potentiated the effect of albumin on increased cytokine release in podocytes. Amount of **A**) IL-6, **B**) TNF, and **C**) IL-1β released into the medium after exposure to 5 mg/mL albumin (white bars), 50 µM chloroquine (grey bars) and 5 mg/mL albumin plus 50 µM chloroquine (black bars). IL-6 (n = 4), TNF (n = 4), and IL-1β (n = 3)were increased in podocytes exposed to both albumin and chloroquine compared to either exposure alone at 18 hrs, *p<0.0001.

As we have shown previously, albumin exposure up-regulates cytokine RNA expression and increases cytokine release in HUPECs, compared to dextran oncotic controls [6]. Whether inhibiting the lysosomal degradation of albumin increases the effect of albumin on cytokine release in podocytes is unknown. After exposing podocytes to culture media alone (control), 50 µM chloroquine in media, 5 mg/ml low endotoxin human albumin alone, or 50 µM chloroquine in 5 mg/ml low endotoxin albumin for 1, 3, or 18 hours, the amount of IL-6, TNF, and IL-1β released into the media was measured by ELISA. The main effect of treatment group was significant as was the effect of time and the treatment group*time interaction for all 3 cytokines. IL-6 was higher in the albumin plus chloroquine group compared to the albumin (p = 0.0385) or chloroquine (p = 0.0005) group alone (**Figure 5A**, n = 4). IL-6 was higher at 18 hours than at 1 or 3 hours (p<0.0001 for both). IL-6 in podocytes exposed to albumin plus chloroquine was higher at 18 hours than any pair-wise comparison (p<0.0001 all). IL-6 in podocytes exposed to albumin for 18 hours was higher than all combinations except exposure to albumin plus chloroquine for 18 hours. TNF was highest in the albumin plus chloroquine group compared to the albumin (p<0.0001) and chloroquine (p<0.0001) group alone (**Figure 5B**, n = 4). At 18 hours TNF was higher than at 1 hour (p<0.0001) and 3 hours (p<0.0001). TNF was highest for podocytes exposed to albumin plus chloroquine at 18 hours compared to all other combinations (p<0.0001). TNF was higher in podocytes exposed to albumin for 18 hours compared to 1 hour (p = 0.0332). For IL-1β albumin was significantly different than albumin plus chloroquine (p<0.0001) but not chloroquine alone (p = 0.0624) (**Figure 5C**, n = 3). IL-1β at 18 hours was significantly higher than 1 hour (p<0.0001) and 3 hours (p<0.0001). Podocytes exposed to albumin plus chloroquine released more IL-1β at 18 hours than any of the other pair-wise comparisons (p<0.0001). Interestingly, the effect of combined albumin and chloroquine exposure on the production of TNF and IL-1β appeared to be greater than the additive effect of exposure to either treatment alone at 18 hours.

### Lysosome inhibition increased podocyte cell death

**Figure 6:**
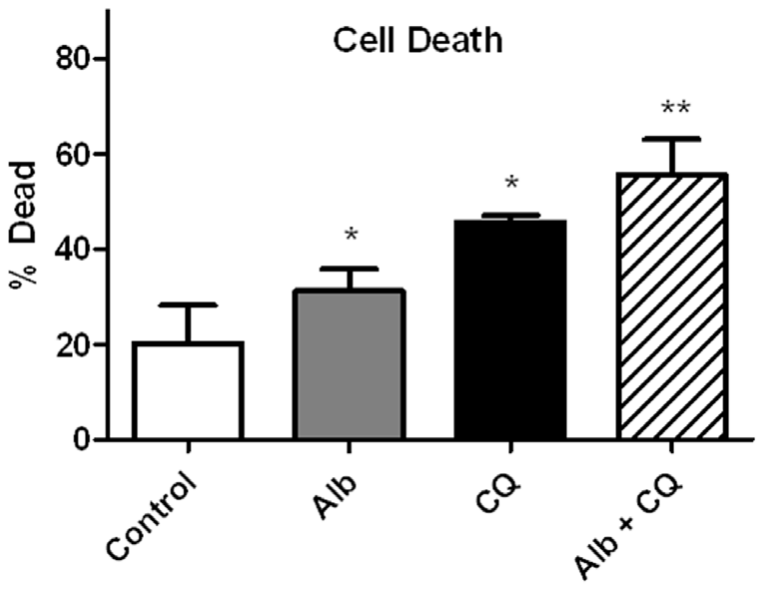
Lysosome inhibition increased podocyte cell death. Shown are the percentages of dead podocyte cells after 24(5 mg/mL), chloroquine (50 µM), and albumin (5 mg/mL) plus chloroquine (50 µM). Compared to control podocytes (n = 5), cell death was increased in albumin (n = 5) and chloroquine (n = 5) exposed podocytes,* p<0.05. Cell death was greater in podocytes exposed to both albumin and chloroquine (n = 5) compared to either albumin or chloroquine and compared to the control, **p<0.05.

Our previous work demonstrates that albumin exposure causes a dose-dependent and time-dependent increase in cell death compared to dextran treated oncotic controls [6]. To determine if lysosome inhibition potentiates the effect of albumin treatment on cell death, HUPECs were exposed to regular media as control, low-endotoxin albumin (5 mg/ml), chloroquine (50 µM), and both albumin and chloroquine combined for 24 hours. Cell death was determined by trypan blue exclusion using an automated cell counter (**Figure 6A**). At 24 h, the percentage of dead cells in the control, albumin exposed, chloroquine exposed, and albumin plus chloroquine exposed groups was 20.2±3.6%, 31.2±2.1%, 45.6±0.6%, and 55.6±3.3%, respectively. Cell death for albumin and chloroquine exposed podocytes was significantly greater than control podocytes (p<0.05). Cell death for podocytes exposed to albumin and chloroquine combined was significantly greater than exposure to either albumin or chloroquine alone (p<0.05).

### Albuminuria, glomerulosclerosis, and lysosomal associated membrane protein 1 (LAMP-1) staining was increased in Denys-Drash mice compared to wild type mice

**Figure 7:**
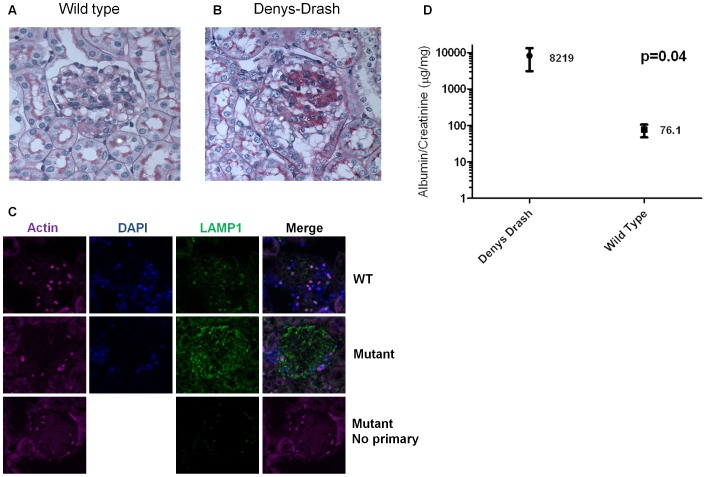
Albuminuria, glomerulosclerosis, and lysosomal associated membrane protein 1 (LAMP-1) staining was increased in Denys-Drash mice compared to control mice. Representative periodic acid-Shiff (PAS) staining of glomeruli from A) wild type and B) Denys-Drash mice. Glomerulosclerosis was qualitatively increased in Denys-Drash mice compared to controls. Representative fluorescence staining for LAMP-1 (green) in glomeruli from C) Denys-Drash and wild type mice. LAMP-1 staining was qualitatively increased in Denys-Drash mice compared to controls. LAMP1 staining appeared to be most prominent in podocytes as demonstrated by the increased intensity at the periphery of the glomerulus. The round bodies within the glomeruli that stain non-specifically for actin and LAMP1 are erythrocytes. E) Urine albumin to creatinine ratios were increased at 4 weeks in Denys-Drash (n = 3) compared to wild type (n = 5) mice. (Shown here on a logarithmic scale as mean ±SEM, p = 0.04).

We used a Denys-Drash mouse model of nephrotic syndrome to determine if lysosomes are altered in nephrotic syndrome *in vivo*. We chose this model of nephrotic disease because it does not involve toxins such as adriamycin or lipopolysaccharide to induce albuminuria. As a result we were able to evaluate the effects of albuminuria itself without the confounding effects of a podocyte toxin. In order to characterize lysosomes in podocytes *in vivo*, sections of renal tissue from Denys-Drash and wild type mice were stained for LAMP-1 at 4 weeks. Glomeruli from Denys-Drash mice (**Figure 7B**) show increased glomerulosclerosis on periodic acid-Shiff (PAS) staining compared to wild type (**Figure 7A**). LAMP-1 (green immunofluorescence) staining of the glomeruli in Denys-Drash mice (**Figure 7C**) was markedly increased compared to wild type (Furthermore, LAMP-1 staining appeared to be most prominent in podocytes as demonstrated by the increased intensity at the periphery of the glomerulus. As expected, albuminuria (**Figure 7D**) was significantly increased in Denys-Drash mice (n = 3) compared to wild type mice (n = 5), 8219±5127 µg albumin/mg creatinine vs 76.1±28.98 µg albumin/mg creatinine, p = 0.04.

## Discussion

One of the primary functions of podocytes is the sieving of macromolecules. Recent studies show podocytes also actively clear the glomerular basement membrane via endocytosis and transcytosis of proteins [36]. With increased protein filtration, via disruption of the endothelial cell layer and/or the glomerular basement membrane, the capacity of podocytes to clear and process proteins is challenged. This is demonstrated by the protein resorption droplets found in podocytes in various nephrotic diseases. We and others have shown that podocytes endocytose albumin [6], [34]. The fate of albumin after it is endocytosed by podocytes is unknown. In order to prevent the accumulation of albumin, podocytes must either degrade the albumin or traffic the albumin back to the cell membrane for transcytosis. The work presented here focuses on the degradation of albumin in podocytes.

Protein degradation is a form of proteolysis that prevents the accumulation of unwanted or abnormal proteins in cells. Intracellular degradation of proteins occurs in either the lysosome or the proteasome. In general, lysosomes non-selectively degrade proteins of intra- or extracellular origin. In contrast, proteasomes degrade intracellular proteins that have been selectively ubiquinated and targeted to the proteasome. Defects in proteasomal [37] and lysosomal [38] degradation of proteins result in a variety of human diseases. Here we present evidence demonstrating that albumin degradation in podocytes occurs primarily in lysosomes.

Lysosomes, first described in 1955 [39], have been primarily regarded as the cell's garbage disposal. Lysosomes are the terminal degradative compartment of endocytic, phagocytic, and autophagic pathways [40]. It is now apparent that lysosomes are involved in many cell processes and are vital to the maintenance of cell homeostasis [38]. Lysosomes play important roles in endocytosis, cholesterol homeostasis, plasma membrane repair, cell death, and autophagy. Autophagy, a major homeostatic process, is entirely reliant on functioning lysosomes. Maintenance of cellular homeostasis is particularly important in podocytes due to their limited ability to regenerate. Hartelben et al. have demonstrated that podocytes possess an unusually high level of constitutive autophagy [41]. The importance of autophagy in podocyte health has been highlighted by several studies in which key regulators of autophagy have been knocked out [41], [42], [43], [44]. Impaired autophagy leads to podocyte cell death which is associated with glomerulosclerosis and predicts renal disease progression [45].

Here we focused directly on lysosomal function in immortalized podocytes from normal subjects. First, we found that endocytosed albumin co-localized with lysosomes in podocytes. We demonstrated that the degradation of endocytosed albumin was inhibited by a lysosomal inhibitor, but not a proteasomal inhibitor. Together these data suggest that albumin degradation in podocytes is lysosomal. We demonstrated that endocytosed albumin increased the density of lysosome fluorescence and potentially impaired lysosomal activity in podocytes. We utilized FRAP to assess lysosome activity. To our knowledge, this is the first study to measure lysosome activity in living podocytes using FRAP. The prolonged t½ of fluorescence recovery reflects a decrease in lysosome activity. However, albumin, like the Magic Red reagent, is a substrate for cathepsin B. Therefore, the prolonged t½ with albumin treatment may represent either an actual decrease in cathepsin B activity, a competitive effect of the albumin for cathepsin B, or both. If the effect of albumin on t½ is one of competitive inhibition of cathepsin B, our hypothesis that excessive albumin uptake impairs lysosome function would not be supported. Nevertheless, it would support the hypothesis that albumin degradation occurs in the lysosome since cathepsin B is only active in lysosomes. By whichever mechanism albumin prolonged t½, the data suggest endocytosed albumin is processed by the lysosome. This interpretation is strongly supported by our data showing that chloroquine, but not MG-132, inhibited albumin degradation in podocytes.

Inhibiting the lysosome with chloroquine exacerbated the negative effect of albumin on lysosome density and activity. Having characterized lysosomal function in podocytes from subjects without glomerular disease, we hope to determine if lysosomal function is impaired in podocytes from subjects with glomerular diseases (e.g. FSGS) in future experiments.

We utilized a Denys-Drash mouse model of nephrotic syndrome to determine if lysosome activity was increased in nephrotic syndrome *in vivo*. We chose this model of nephrotic disease because it does not involve toxins such as adriamycin or lipopolysaccharide to induce albuminuria. As a result we were able to evaluate the effects of albuminuria itself without the confounding effects of a podocyte toxin. We demonstrated that glomerular staining of the lysosomal marker, LAMP-1, was increased in DD mice, but not wild type mice. As expected, the DD mice exhibited increased glomerulosclerosis and proteinuria compared to wild type. While our data demonstrate an association between increased proteinuria and LAMP-1 staining, we are unable to conclude that the increased proteinuria directly altered lysosome activity in podocytes in vivo. Due to technical limitations encountered in attempts to stain the histologic sections for albumin, we were unable to show co-localization of albumin and LAMP-1 in vivo. Nevertheless, a direct relationship, in vivo, between proteinuria and lysosome activity in podocytes is plausible and is a hypothesis which should be pursued further.

In previous work, we and other investigators have found that albumin itself is toxic to podocytes [5], [6], [7], [8]. In our previous work, we examined the effects of increasing the amount of albumin to which podocytes are exposed to approximately 8 mg/ml (3 mg/ml BSA +5 mg/ml endotoxin free human albumin). This amounts to approximately 2.5 times the amount of albumin found in media supplemented with 10% FBS alone. Previously, we found that podocytes treated with media +10% FBS+5 mg/ml dextran (as an oncotic control) did not have any increase in cytokine production. Thus, we believe that the increase in cytokine production seen with the addition of albumin is due to the effects of an increase in albumin concentration.

In these experiments we showed that the toxic effects of albumin on podocytes were potentiated by lysosomal inhibition. This is reflected by increased cytokine production and cell death in podocytes exposed to albumin and chloroquine combined compared to either treatment alone. The direct link between albumin accumulation and cell toxicity is unknown. Autophagy, which is critical to podocyte function [41] has been shown to suppress cytokine expression [46], [47]. This may explain the increase in cytokine expression with inhibition of lysosomes, which are vital to autophagy. One may speculate that overwhelming the lysosome with accumulating albumin impairs the ability of the lysosome to perform other vital functions, thereby disrupting cell homeostasis. Lysosome membranes separate an acidic environment and hydrolases, specifically cathepsins, from the cell cytoplasm. Permeabilization of lysosomal membranes results in leakage of harmful protons and cathepsins into the cytoplasm leading to apoptosis and necrosis [48]. The intracellular accumulation of proteins may result in the disruption of lysosomal membranes and leakage of enzymes into the cytoplasm as suggested by earlier studies on renal tubular cells [49], [50], [51]. Whether or not the accumulation of albumin in podocyte lysosomes results in permeabilization of the lysosome membrane is unknown.

It is possible that the toxic effects of albumin and chloroquine are independent of one another. However, the effect of the combined exposure of albumin plus chloroquine on TNF and IL-1β production was more than an additive effect. This suggests the effects of albumin and chloroquine may be potentially synergistic. It is also possible that the toxic effects of chloroquine on cells may be independent of its effects on the lysosome. However, we used a concentration of chloroquine that is well tolerated by other cell types [52]. Hydroxychloroquine has been shown in case reports to be toxic to podocytes, causing an iatrogenic phospholipidosis which mimics Fabry's disease [53]. The toxicity of hydroxychloroquine in this incidence, however, is through its inhibitory effects on lysosomal enzymes.

There are several examples of lysosome dysfunction leading to human disease. In the kidney, lysosome dysfunction causes Fabry disease and cystinosis. The glomerular lesions in Fabry disease include hypertrophy of podocytes, which accumulate foamy appearing vacuoles, and a progression from segmental to global glomerulosclerosis [54]. While the major effect of cystinosis is tubular dysfunction, podocytopathy marked by foot process fusion and focal segmental glomerulosclerosis has been reported [55]. Interestingly, in the aforementioned studies investigating the role of autophagy on podocyte health, vacuolization of podocytes and progressive glomerulosclerosis were lesions common to all models. This suggests lysosome impairment may be a common pathological pathway to the development of focal segmental glomerulosclerosis.

Elucidation of the role of lysosomes in disease has identified new therapeutic targets, particularly in lysosomal storage diseases and neurodegenerative diseases [38], [56]. For example, Z-Phe-Ala-diazomethylketone (PADK), a mild inhibitor of cathepsin B and L, has been shown to enhance hydrolase synthesis and maturation in lysosomes, thereby effectively increasing the degradative capacity of the lysosome [57]. PADK administered systemically in a mouse model of Alzheimer's disease increased cathepsin B expression and activity, and resulted in the clearance of amyloid beta within neurons [58]. PADK could potentially decrease the accumulation of protein within podocytes that appears to occur in certain glomerular diseases, particularly the collapsing variant of FSGS. There are a number of other inhibitors and activators of lysosomes and autophagy which may have the therapeutic potential [59].

Evidence supporting the importance of autophagy, and therefore lysosomal function, in podocyte homeostasis and glomerular disease is growing [41], [42], [43], [44], [60], [61], [62]. Here we showed that podocytes endocytosed albumin and degraded albumin in lysosomes. Directly inhibiting lysosomes and decreasing the capacity of podocytes to process and degrade albumin increased podocyte inflammation and cell death. Modifiers of lysosomal activity may have therapeutic potential in slowing the progression of glomerulosclerosis by enhancing the ability of podocytes to process and degrade albumin.

## Supporting Information

Data S1 **Leupeptin Data.**
(TIF)
